# Supplementary material for: Escherichia coli Nissle 1917 administered as a dextranomar microsphere biofilm enhances immune responses against human rotavirus in a neonatal malnourished pig model colonized with human infant fecal microbiota
Source: PLoS One. 2021 Feb 16;16(2):e0246193. doi: 10.1371/journal.pone.0246193 (PMC7886176; doi:10.1371/journal.pone.0246193)
Supplement: S1 Fig — (PPTX) [file pone.0246193.s001.pptx]

## Slide 1
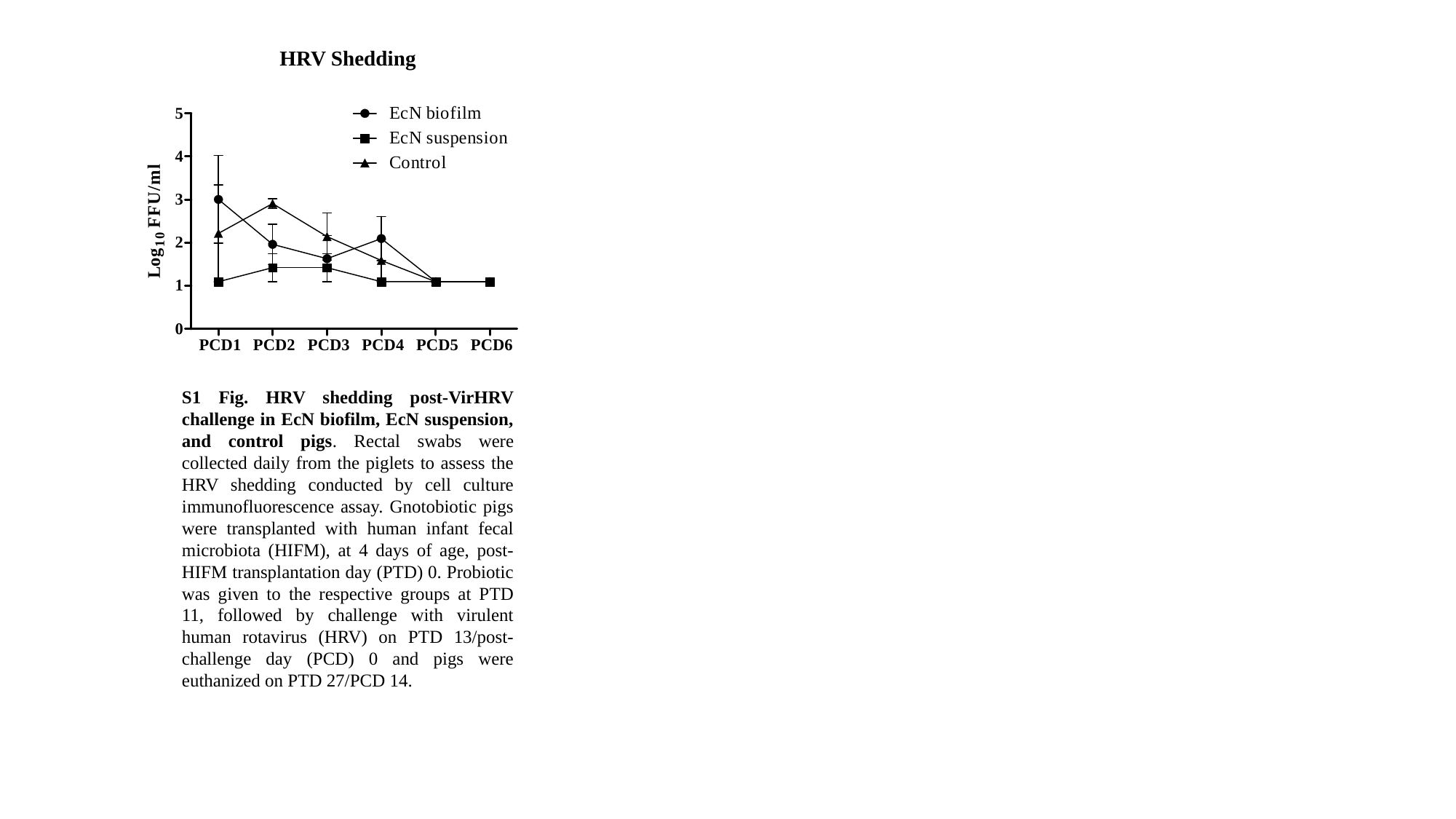

HRV Shedding
S1 Fig. HRV shedding post-VirHRV challenge in EcN biofilm, EcN suspension, and control pigs. Rectal swabs were collected daily from the piglets to assess the HRV shedding conducted by cell culture immunofluorescence assay. Gnotobiotic pigs were transplanted with human infant fecal microbiota (HIFM), at 4 days of age, post-HIFM transplantation day (PTD) 0. Probiotic was given to the respective groups at PTD 11, followed by challenge with virulent human rotavirus (HRV) on PTD 13/post-challenge day (PCD) 0 and pigs were euthanized on PTD 27/PCD 14.
