# Supplementary material for: Escherichia coli Nissle 1917 administered as a dextranomar microsphere biofilm enhances immune responses against human rotavirus in a neonatal malnourished pig model colonized with human infant fecal microbiota
Source: PLoS One. 2021 Feb 16;16(2):e0246193. doi: 10.1371/journal.pone.0246193 (PMC7886176; doi:10.1371/journal.pone.0246193)
Supplement: S2 Fig — (PPTX) [file pone.0246193.s002.pptx]

## Slide 1
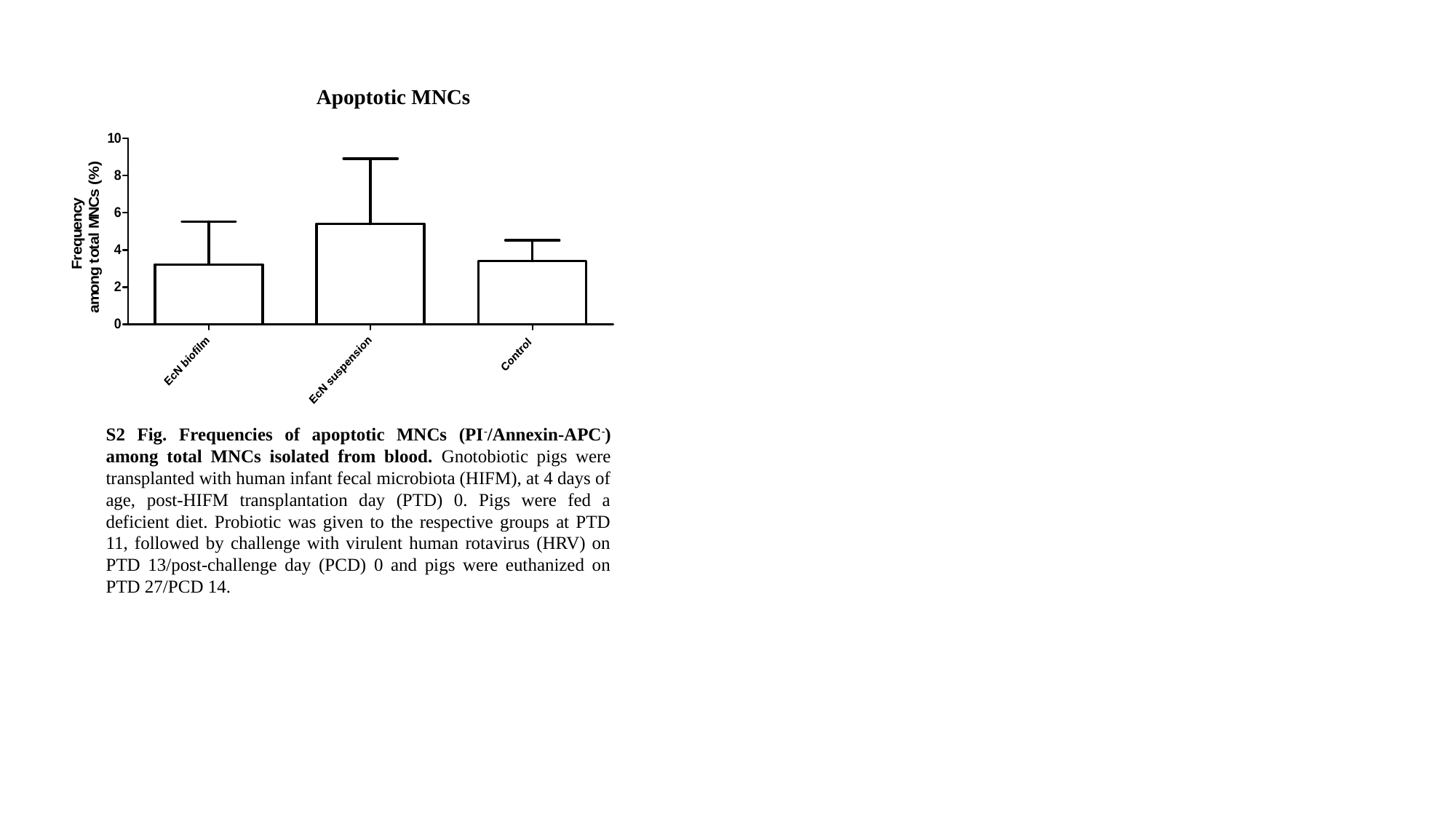

Apoptotic MNCs
S2 Fig. Frequencies of apoptotic MNCs (PI-/Annexin-APC-) among total MNCs isolated from blood. Gnotobiotic pigs were transplanted with human infant fecal microbiota (HIFM), at 4 days of age, post-HIFM transplantation day (PTD) 0. Pigs were fed a deficient diet. Probiotic was given to the respective groups at PTD 11, followed by challenge with virulent human rotavirus (HRV) on PTD 13/post-challenge day (PCD) 0 and pigs were euthanized on PTD 27/PCD 14.
