# Supplementary material for: Escherichia coli Nissle 1917 administered as a dextranomar microsphere biofilm enhances immune responses against human rotavirus in a neonatal malnourished pig model colonized with human infant fecal microbiota
Source: PLoS One. 2021 Feb 16;16(2):e0246193. doi: 10.1371/journal.pone.0246193 (PMC7886176; doi:10.1371/journal.pone.0246193)
Supplement: S4 Fig — (PPTX) [file pone.0246193.s004.pptx]

## Slide 1
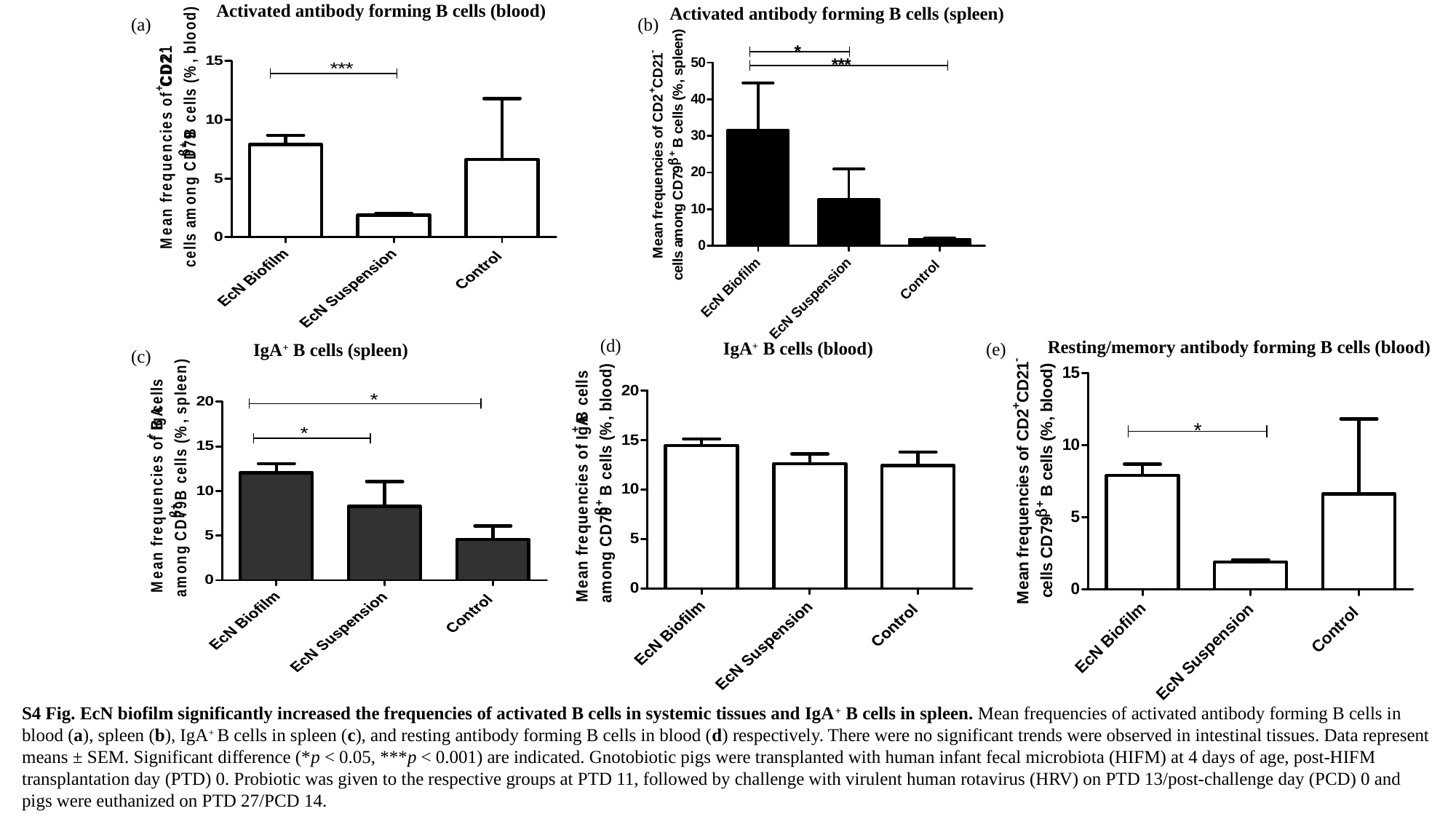

Activated antibody forming B cells (blood)
Activated antibody forming B cells (spleen)
(b)
(a)
(d)
Resting/memory antibody forming B cells (blood)
IgA+ B cells (blood)
(e)
IgA+ B cells (spleen)
(c)
S4 Fig. EcN biofilm significantly increased the frequencies of activated B cells in systemic tissues and IgA+ B cells in spleen. Mean frequencies of activated antibody forming B cells in blood (a), spleen (b), IgA+ B cells in spleen (c), and resting antibody forming B cells in blood (d) respectively. There were no significant trends were observed in intestinal tissues. Data represent means ± SEM. Significant difference (*p < 0.05, ***p < 0.001) are indicated. Gnotobiotic pigs were transplanted with human infant fecal microbiota (HIFM) at 4 days of age, post-HIFM transplantation day (PTD) 0. Probiotic was given to the respective groups at PTD 11, followed by challenge with virulent human rotavirus (HRV) on PTD 13/post-challenge day (PCD) 0 and pigs were euthanized on PTD 27/PCD 14.
