# Supplementary material for: Escherichia coli Nissle 1917 administered as a dextranomar microsphere biofilm enhances immune responses against human rotavirus in a neonatal malnourished pig model colonized with human infant fecal microbiota
Source: PLoS One. 2021 Feb 16;16(2):e0246193. doi: 10.1371/journal.pone.0246193 (PMC7886176; doi:10.1371/journal.pone.0246193)
Supplement: S5 Fig — (PPTX) [file pone.0246193.s005.pptx]

## Slide 1
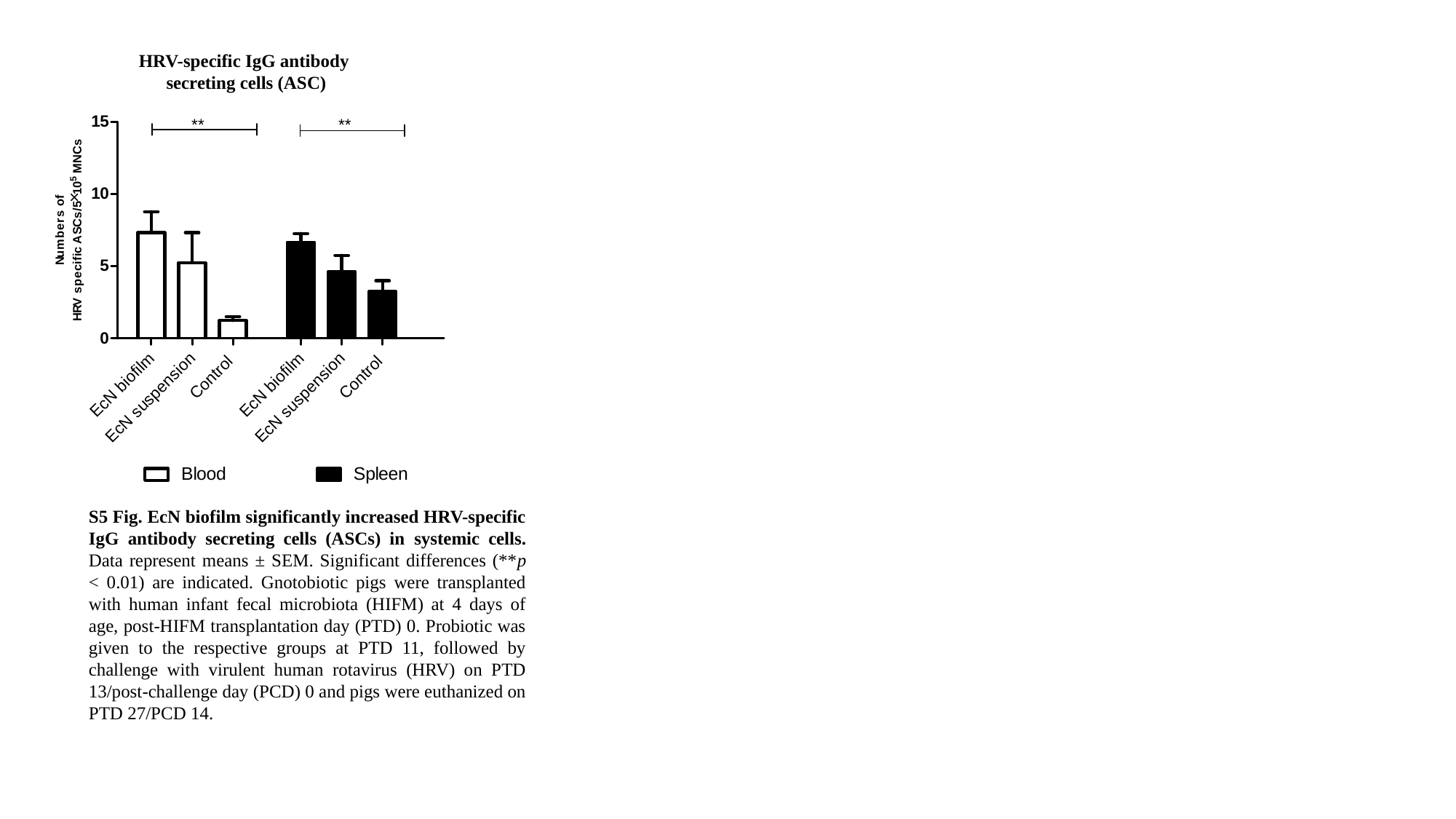

HRV-specific IgG antibody
secreting cells (ASC)
S5 Fig. EcN biofilm significantly increased HRV-specific IgG antibody secreting cells (ASCs) in systemic cells. Data represent means ± SEM. Significant differences (**p < 0.01) are indicated. Gnotobiotic pigs were transplanted with human infant fecal microbiota (HIFM) at 4 days of age, post-HIFM transplantation day (PTD) 0. Probiotic was given to the respective groups at PTD 11, followed by challenge with virulent human rotavirus (HRV) on PTD 13/post-challenge day (PCD) 0 and pigs were euthanized on PTD 27/PCD 14.
