# Supplementary material for: Escherichia coli Nissle 1917 administered as a dextranomar microsphere biofilm enhances immune responses against human rotavirus in a neonatal malnourished pig model colonized with human infant fecal microbiota
Source: PLoS One. 2021 Feb 16;16(2):e0246193. doi: 10.1371/journal.pone.0246193 (PMC7886176; doi:10.1371/journal.pone.0246193)
Supplement: S6 Fig — (PPTX) [file pone.0246193.s006.pptx]

## Slide 1
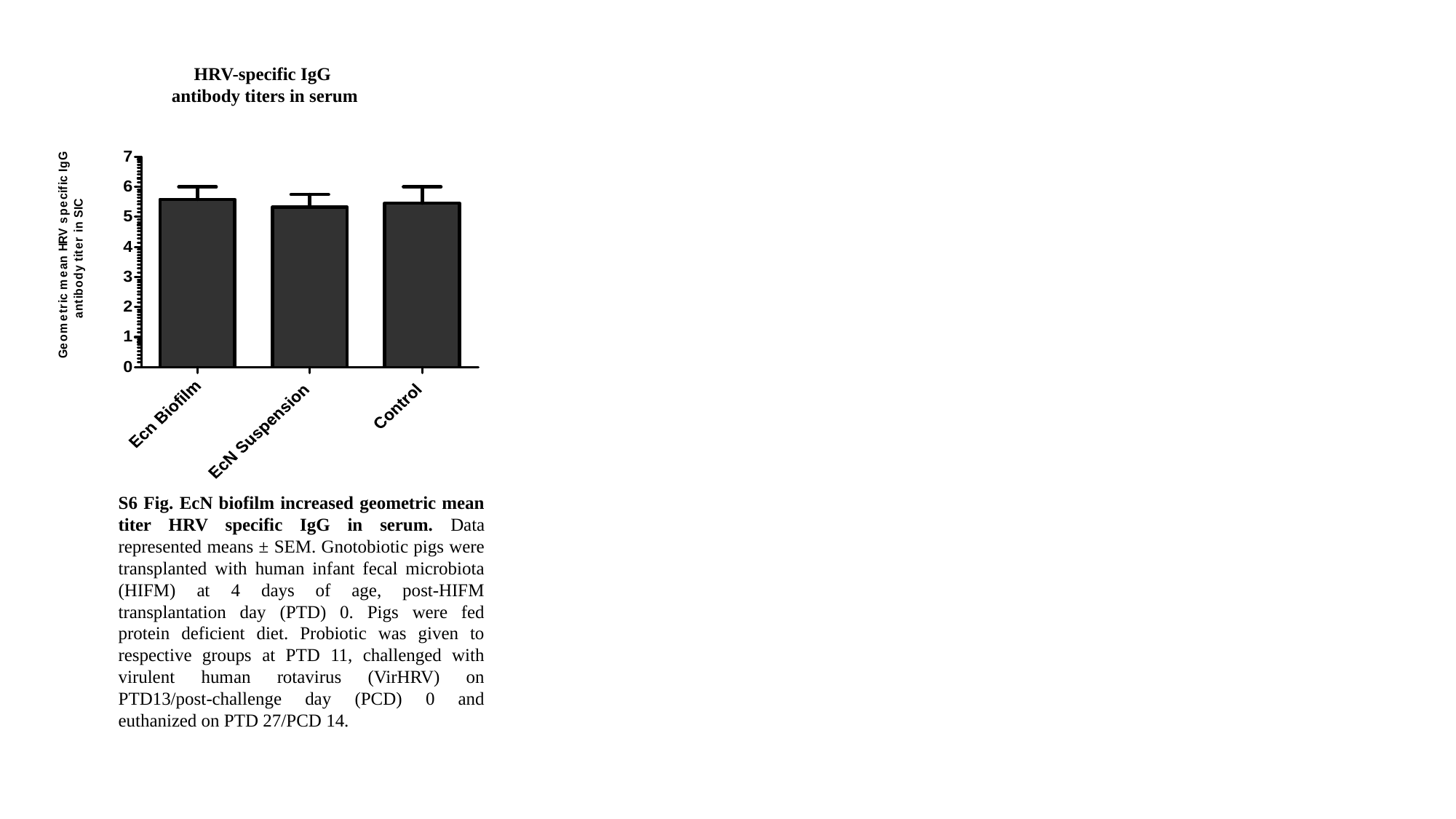

HRV-specific IgG
antibody titers in serum
S6 Fig. EcN biofilm increased geometric mean titer HRV specific IgG in serum. Data represented means ± SEM. Gnotobiotic pigs were transplanted with human infant fecal microbiota (HIFM) at 4 days of age, post-HIFM transplantation day (PTD) 0. Pigs were fed protein deficient diet. Probiotic was given to respective groups at PTD 11, challenged with virulent human rotavirus (VirHRV) on PTD13/post-challenge day (PCD) 0 and euthanized on PTD 27/PCD 14.
