# Supplementary material for: Escherichia coli Nissle 1917 administered as a dextranomar microsphere biofilm enhances immune responses against human rotavirus in a neonatal malnourished pig model colonized with human infant fecal microbiota
Source: PLoS One. 2021 Feb 16;16(2):e0246193. doi: 10.1371/journal.pone.0246193 (PMC7886176; doi:10.1371/journal.pone.0246193)
Supplement: Attachment — Submitted filename: ResponseToReviewer (final).docx [file pone.0246193.s010.docx]

12.29.2020

Manuscript ID: PONE-D-20-32708

Dear Editor,

We sincerely thank you for the opportunity to re-submit our revised manuscript and the reviewer for the constructive and insightful criticism and the overall encouraging comments. Please find below our point-by-point answers to the reviewer’s queries and modifications/insertions are highlighted in the manuscript.

**ED:** Please ensure that your manuscript meets PLOS ONE's style requirements, including those for file naming. The PLOS ONE style templates can be found at https://journals.plos.org/plosone/s/file?id=wjVg/PLOSOne_formatting_sample_main_body.pdf and https://journals.plos.org/plosone/s/file?id=ba62/PLOSOne_formatting_sample_title_authors_affiliations.pdf

**AU:** We have reviewed the PLOS ONE guidelines and believe our manuscript meets them.

**ED:** Please provide additional details regarding participant consent. In the ethics statement in the Methods and online submission information, please ensure that you have specified what type you obtained (for instance, written or verbal, and if verbal, how it was documented and witnessed). If your study included minors, state whether you obtained consent from parents or guardians. If the need for consent was waived by the ethics committee, please include this information.

**AU:** We have obtained a written consent from parent that is being securely stored.

**ED:** Thank you for stating the following in the Acknowledgments Section of your manuscript:

"This work was supported by the Bill and Melinda Gates Foundation (OPP 1117467), the NIAID,

NIH (R01 A1099451), federal and state funds appropriated to the Ohio Agricultural Research and

Development Center, The Ohio State University and from the NIH Office of Dietary Supplements

(ODS) supplemental grant funds."

We note that you have provided funding information that is not currently declared in your Funding Statement. However, funding information should not appear in the Acknowledgments section or other areas of your manuscript. We will only publish funding information present in the Funding Statement section of the online submission form.

Please remove any funding-related text from the manuscript and let us know how you would like to update your Funding Statement. Currently, your Funding Statement reads as follows:

"NO: The funders had no role in study design, data collection and analysis, decision to publish, or preparation of the manuscript."

Please include your amended statements within your cover letter; we will change the online submission form on your behalf.

AU: Funding information is removed from the manuscript. During the original manuscript submission, we have entered the funding sources; however, we failed to find where Funding statement could be entered. Please enter the following statement into the online submission system:

This work was supported by the Bill and Melinda Gates Foundation (OPP 1117467), the NIAID, NIH (R01 A1099451), federal and state funds appropriated to the Ohio Agricultural Research and Development Center, The Ohio State University and from the NIH Office of Dietary Supplements (ODS) supplemental grant funds.

**ED:** We note that you have included the phrase “data not shown” in your manuscript. Unfortunately, this does not meet our data sharing requirements. PLOS does not permit references to inaccessible data. We require that authors provide all relevant data within the paper, Supporting Information files, or in an acceptable, public repository. Please add a citation to support this phrase or upload the data that corresponds with these findings to a stable repository (such as Figshare or Dryad) and provide and URLs, DOIs, or accession numbers that may be used to access these data. Or, if the data are not a core part of the research being presented in your study, we ask that you remove the phrase that refers to these data.3.

AU: To comply with the PLOS policy we have now submitted all the data previously labeled as ‘not shown’ as supplementary figures and numbered accordingly.

Reviewer # 1 Comments to the Author

RE: Lines 84-87: Please consider to include in this section some information about microparticles and materials studied or proved useful to improve probiotics viability (as explanation of the hypothesis).

AU: We have now added the following information: Navarro and his colleagues (2017) have formulated a new synbiotic formulation that employed porous semi-permeable, biocompatible and biodegradable microspheres (dextranomer microspheres) containing readily diffusible prebiotic cargo. Adherence of the probiotic bacteria to the microsphere has a two-fold effect; it facilitates the more formidable biofilm state of probiotics as well as a creates a directed means to provide a high concentration gradient of prebiotics via diffusion of the microsphere cargo.

Now lines 85-91.

RE: Lines 88-100: Besides gut stability, Why a single administration (EcN-biofilm) would be considered enough?

AU: Biofilms are the native stable state of bacteria on surfaces and provide a more protected state of the resident bacteria. They also allow bacteria to evade innate immune defenses and persist in the gut longer (Tam, Uyen et al., 2006; Wang, Cao et al. 2020). These biofilm features allow maximization of the beneficial effects of probiotics following single administration.

RE: Lines 101-105: In some cases, with the use of probiotics is recommended the use of prebiotics and supplements (to restore gut balance). Explain better the impact of malnutrition in diarrheic diseases and the limitations of the probiotics’ beneficial effects.

AU: Malnutrition increases the risk of diarrheal diseases caused by some, but not all, entero-pathogens. Malnutrition can result in impaired immune defenses that compromise gut integrity, and dybiosis that can influence defense against intestinal pathogens in the malnourished host.

Now lines 108-111.

Probiotics are generally considered safe, however there are some associated risks. These risks are increased if there are chronic medical conditions that weaken the immune system or if there are gut barrier breeches. Possible risks can include: developing an infection, developing resistance to antibiotics, and developing harmful byproducts from the probiotic supplement. Also, in malnourished hosts due to increased intestinal motility, probiotics can be eliminated from the gut faster limiting their beneficial effects.

Now lines 114-119.

RE: Lines 321-323: Although EcN biofilm showed better results, EcN suspension also had good response controlling viral shedding respect to control (and in other parameters). Is the cost-effect good or better in the use of EcN biofilm (production)?

AU: Results suggest that low-cost, stable, and efficient dietary supplementation of EcN coupled with a dextranomer microsphere biofilm can protect against HRV infection and can moderate the symptoms of other gastrointestinal infections and disorders including gastritis and chronic inflammatory bowel disease. Probiotics in the planktonic state need to be taken daily, whereas this biofilm formulation only needs to be taken once which can reduce the cost, despite the extra expense for microsphere formulation.

RE: Lines 123-125: Why did you decided to use HRV strain Wa instead OSU? Is just because the gut microbiota modification?

AU: This is because we were modeling childhood malnutrition-rotavirus infection-probiotic intervention in gnotobiotic pigs transplanted with human infant microbiota and infected with human rotavirus strain (Wa).

RE: Line 393: more than speculate use other expression… change it by -this would explain- or -this agreed with our hypothesis-

AU: Word ‘speculate’ is replaced with ‘this agreed with our hypothesis’ and now line 395.

RE: References: Check the references format; in reference 13, the year was missed. Most of the references are old (20% are from 2015-2020)

AU: Year is added into the reference, now line 503. Older references including the primary references can be highly relevant—our focus was to cite the most relevant references!
